# Development and validation of a 4-color multiplexing spinal muscular atrophy (SMA) genotyping assay on a novel integrated digital PCR instrument

**DOI:** 10.1038/s41598-020-76893-7

**Published:** 2020-11-16

**Authors:** Lingxia Jiang, Robert Lin, Steve Gallagher, Andrew Zayac, Matthew E. R. Butchbach, Paul Hung

**Affiliations:** 1 Combinati Inc., 2450 Embarcadero Way, Palo Alto, CA 94303 USA; 2 Center for Applied Clinical Genomics, Nemours Biomedical Research, Nemours Alfred I. duPont Hospital for Children, Wilmington, DE USA; 3 Center for Pediatric Research, Nemours Biomedical Research, Nemours Alfred I. duPont Hospital for Children, Wilmington, DE USA; 4 Department of Pediatrics, Sidney Kimmel College of Medicine, Thomas Jefferson University, Philadelphia, PA USA; 5 Department of Biological Sciences, University of Delaware, Newark, DE USA

**Keywords:** Biotechnology, Genetics, Molecular biology, Diseases

## Abstract

Digital PCR (dPCR) technology has been proven to be highly sensitive and accurate in detecting copy number variations (CNV). However, a higher-order multiplexing dPCR assay for measuring *SMN1* and *SMN2* copy numbers in spinal muscular atrophy (SMA) samples has not been reported. Described here is a rapid multiplex SMA dPCR genotyping assay run on a fully integrated dPCR instrument with five optical channels. The hydrolysis probe-based multiplex dPCR assay quantifies *SMN1*, *SMN2,* and the total *SMN* (*SMN1* + *SMN2*) while using *RPPH1* gene as an internal reference control. The quadruplex assay was evaluated with characterized control DNA samples and validated with 15 blinded clinical samples from a previously published study. *SMN1* and *SMN2* copy numbers were completely concordant with previous results for both the control and blinded samples. The dPCR-based SMA copy number determination was accomplished in 90 min with a walk-away workflow identical to real-time quantitative PCR (qPCR). In summary, presented here is a simple higher-order multiplexing solution on a novel digital PCR platform to meet the growing demand for SMA genotyping and prognostics.

## Introduction

Proximal spinal muscular atrophy (SMA) is an early-onset neurodegenerative disease characterized by the loss of α-motor neurons in the anterior horn of the spinal cord which leads to muscle weakness and atrophy^1,2^. SMA has an autosomal recessive inheritance pattern with an incidence of 1 in 6000–10,000 births^3,4^. SMA results from the loss or mutation of *survival motor neuron 1* (*SMN1)* on the q arm (q13) of chromosome *5* and retention of the paralogous *survival motor neuron 2* (*SMN2*)^5^. *SMN1* and *SMN2* are nearly identical save 5 key nucleotide differences at the 3′ ends of the genes. *SMN2* is functionally distinguishable from *SMN1* by a single nucleotide difference (c.840C>T) in exon 7 that disrupts an exonic splice enhancer^6,7^. Even though close to 95% of SMA cases result from the loss of *SMN1* and retention of *SMN2*, there is a wide spectrum of clinical disease severity based on the motor development milestones that are achieved^8^. Numerous studies using different assays and patient cohorts (reviewed in^9^) have demonstrated a strong inverse correlation between *SMN2* copy number and disease severity. There are currently three USA Food and Drug Administration (FDA) approved therapeutic options; nusinersen (Spinraza, Biogen), onasemnogene abeparvovec (Zolgensma, AveXis/Novartis) and risdiplam (Evrysdi, Roche)^10–14^. These agents target *SMN2* expression. *SMN2* copy number is used to design the therapeutic regimens for SMA patients receiving these approved therapies, with immediate treatment for patients with 2 or 3 copies of *SMN2*^15,16^. A simple yet highly accurate and sensitive solution for *SMN1* and *SMN2* copy number detection is needed to meet the rapidly growing demand for informed SMA treatment.

Current genotyping and copy number determination methods for SMA are complex, time consuming or lack resolution with higher copy numbers. Multiplex ligation-dependent probe amplification (MLPA) and real-time quantitative PCR (qPCR) can detect *SMN1* deletions^17–20^. MLPA is a multi-step method requiring post-PCR capillary electrophoresis and a long time-to-result (~ 20 h)^18,19^. Alternatively, qPCR requires normalization with standard curves. More importantly, neither MLPA nor qPCR can consistently distinguish unit differences in *SMN1* or *SMN2* when the copy number is greater than 3^20–22^. Genomic sequencing (GS) technology has made progress on copy number detection. A recent publication reported SMA diagnosis and carrier identification using whole genome sequencing and advanced copy number analysis software^23^. However, there are still limitations to GS for diagnosis including the cost, sample processing time, complex data analysis and the need for orthogonal validation.

**Figure 1:**
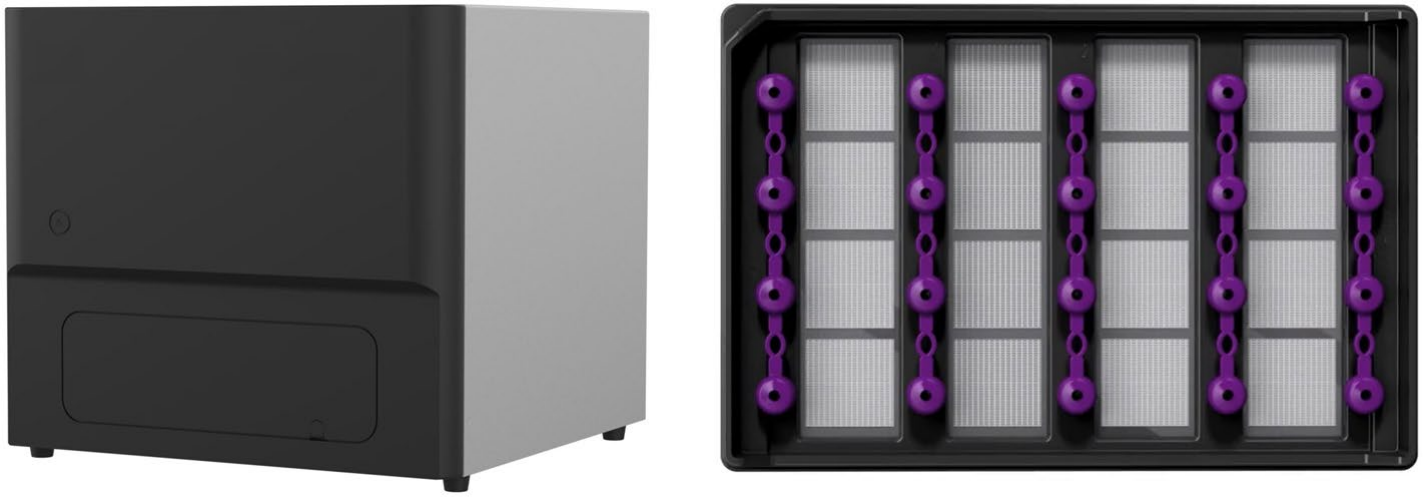
Components of the Combinati Absolute Q Digital PCR System with a 16-sample microfluidic array partitioning (MAP) plate. The instrument integrates thermal systems for PCR, pneumatic systems for reagent digitization and optical systems for raw data acquisition.

Digital PCR (dPCR) alleviates the limitations previously noted to provide absolute quantification of a target gene within a sample for molecular diagnostics and prognostics^24^. With dPCR, the prepared PCR sample is distributed across a large number of physically isolated micro-reaction partitions so that each partition will have single digit counts of template DNA or none at all^25,26^. The absolute concentration (and confidence level) of the target gene(s) can be calculated by counting the number of positive partitions (containing at least one target molecule) and the number of negative partitions (containing no target molecules) and using a Poisson-based statistical correction. dPCR can reliably and accurately measure *SMN1* and *SMN2* copy numbers over a wide range, between 0 and 6 copies^26,27^. Due to the optical configuration of first-generation dPCR platforms, most of the currently available multiplex assays are duplex or variations of a duplex assay. A recently published report on multiplex dPCR describes simultaneous identification of *SMN1* and *SMN2* copy numbers by using different signal amplitudes within the same optical channel^28^. The Combinati (Palo Alto, California) Absolute Q Digital PCR System (Fig. 1) is a single benchtop instrument with 5 optical channels and a qPCR-equivalent, walkaway workflow^29^. Instead of generating air and water emulsions, the Absolute Q features rigid micro-injection-molded partitioning consumable plates which ensure highly consistent partition yield and volume. In this study, the Absolute Q is used to demonstrate the first “higher order” multiplex dPCR assay for simultaneous SMA diagnostic, i.e. loss of *SMN1*, and prognostic, i.e. *SMN2* copy number, assessment.

## Results

### Multiplex SMA genotyping assay

**Figure 2:**
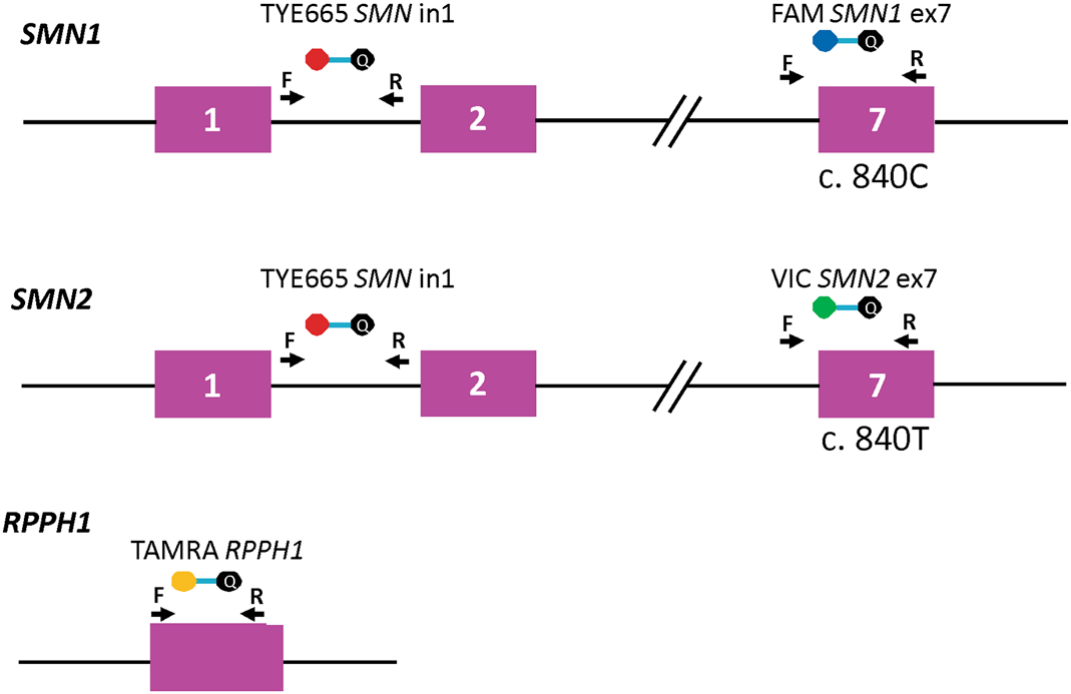
Schematic representation of the 4-color multiplex assay design showing the positions of the 4 probes. Arrows represent forward and reverse primers of each assay. The bars connecting two circles represent probes while the color-filled circles represent different fluorophores and the black circles represent dark quenchers. The dashed lines (//) indicate discontinuous sequence.

The hydrolysis probe-based multiplex assay is designed to use FAM, VIC, TYE665, and TAMRA to identify *SMN1*, *SMN2*, total *SMN* (*SMN1* + *2*), and *RPPH1,* respectively (Fig. 2). The *SMN1* probe (FAM) hybridizes to nucleotide c.840C while the *SMN2* probe (VIC) hybridizes to nucleotide c.840T. The total *SMN* (*SMN1* + *2,* TYE665) targets intron 1—a region that is identical between *SMN1* and *SMN2*. The internal reference control is designed to target the highly conserved *RPPH1* gene (TAMRA), which is consistently present as 2 copies^30^.

**Figure 3:**
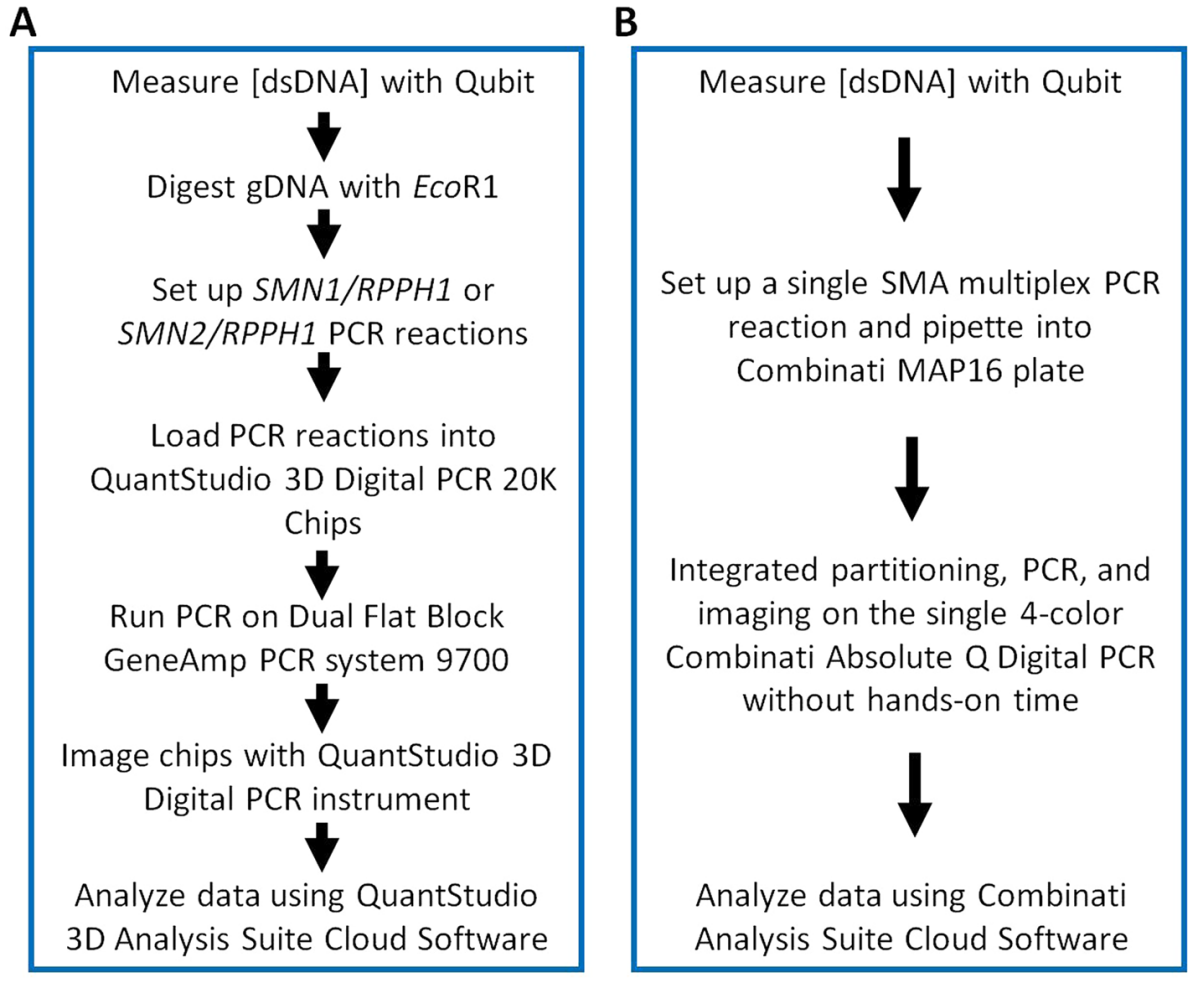
Comparison of the assay workflow for the QuantStudio 3D dPCR System (**A**; ^27^) and the Combinati Absolute Q dPCR System (**B**). Panel A is reproduced from^27^ with permission from the authors (2015) in accordance with Creative Commons license CC BY 3.0.

The workflow with the Absolute Q dPCR System involved fewer steps when compared to the *SMN1* and *SMN2* copy number assays previously developed on the QuantStudio 3D dPCR System (Fig. 3)^27^. As the components of the dPCR assay are integrated into a single unit, the Combinate Absolute Q dPCR assay required less hands-on time for the operator. Furthermore, the multiplex SMA genotyping assay was accomplished in 90 min on the Absolute Q dPCR System. The *SMN1* and *SMN2* copy number analysis was completed in 5.5 h on the QuantStudio 3D dPCR System^27^.

**Figure 4:**
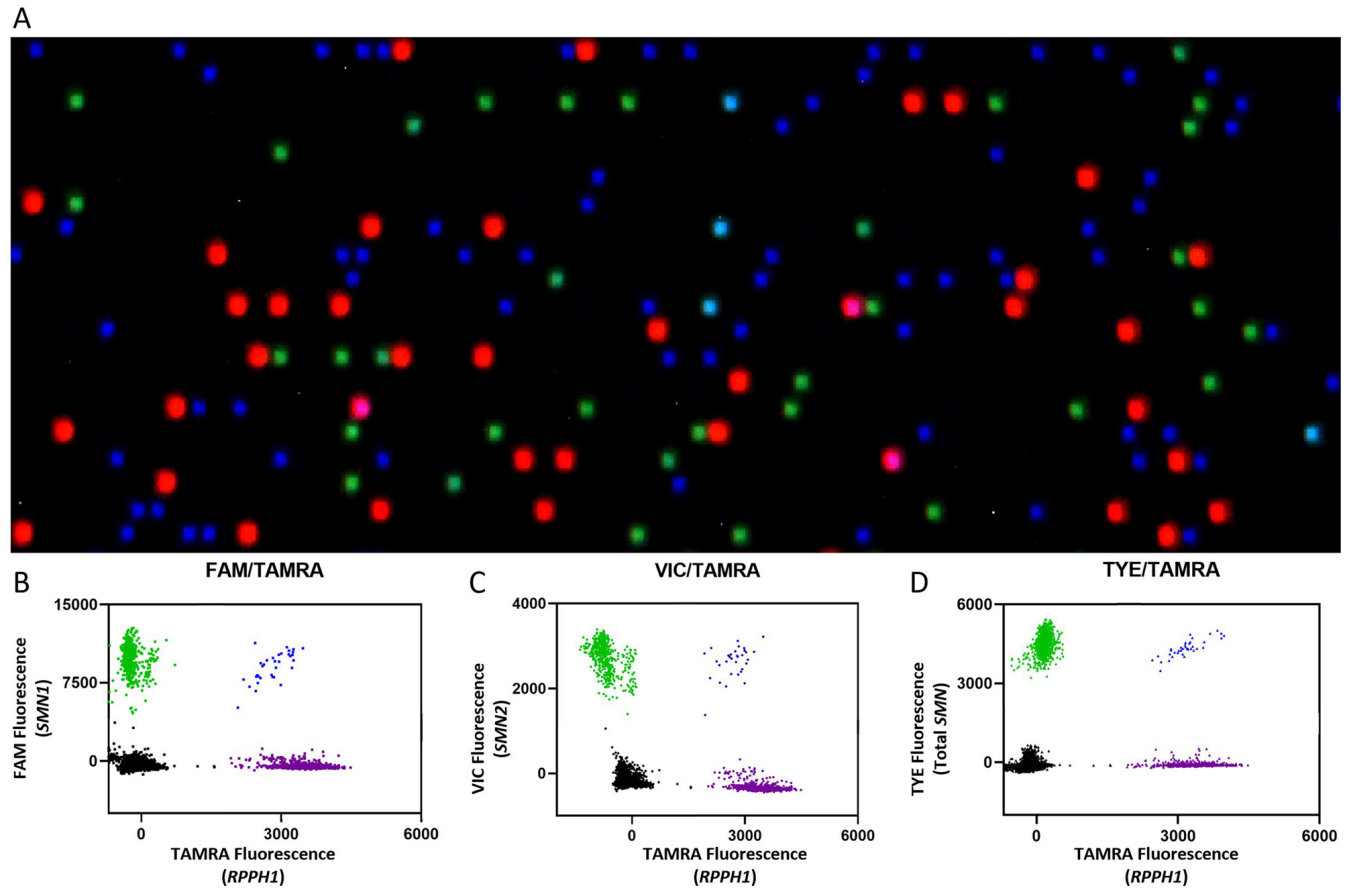
Representative partition image (**A**) and scatter plots (**B**–**D**) of the SMA 4-color multiplexing dPCR assay using NA03815 genomic DNA as the sample. The scatter plots are shown for *SMN1* exon 7 (**B**; FAM), *SMN2* exon 7 (**C**; VIC) and total *SMN* intron 1 (**D**; TYE665), all relative to the reference gene *RPPH1* (TAMRA). The analysis software subtracted the pre-PCR image intensities from the post-PCR image intensities so as to remove fluorescent signals which did not exhibit amplification behavior, thereby eliminating false positive signals. If the pre-PCR partitions were brighter than the post-PCR partitions, then the analysis software would record negative fluorescent units. These negative units, however, did not impact the copy number quantification results.

The optimized assay was first tested on control DNA samples containing *SMN1* only (NA17117), *SMN2* only (NA23255), or both *SMN1* and *SMN2* (NA03815). Representative images of the dPCR results from the analysis software (Fig. 4A) shows strong resolution of the partitions. All SMN probes (*SMN1* exon 7, *SMN2* exon 7 and total *SMN* intron 1) were normalized against the reference gene (*RPPH1*) that contains 2 copies per genome. The scatter plots for *SMN1* exon 7 (Fig. 4B), *SMN2* exon 7 (Fig. 4C), and total *SMN* intron 1 (Fig. 4D) shows robust separation between the target and reference probes. The multiplex SMA genotyping assay run on the Absolute Q shows high specificity and sensitivity in classifying the control samples with *SMN1*:*SMN2* copy numbers of 0:3 (NA23255), 3:0 (NA17117), and 1:1 (NA03815). For NA03815, the Absolute Q dPCR data showed copy numbers for *SMN1* exon 7, *SMN2* exon 7, and total *SMN* intron 1 as 1.1, 1.1 and 2.2, respectively.

The Absolute Q dPCR assays require less template genomic DNA (2.5 ng) than the QuantStudio 3D dPCR assays which uses 30–60 ng of *Eco*RI-digested genomic DNA. Genomic DNA up to 25 ng was also tested on the Absolute Q. There are no apparent differences in *SMN1* and *SMN2* copy number quantification completed with 2.5 ng template or with 25 ng genomic DNA (Supplementary Table S1).

### Quantification of control gDNA *SMN1* and *SMN2* copy numbers using absolute Q dPCR

**Figure 5:**
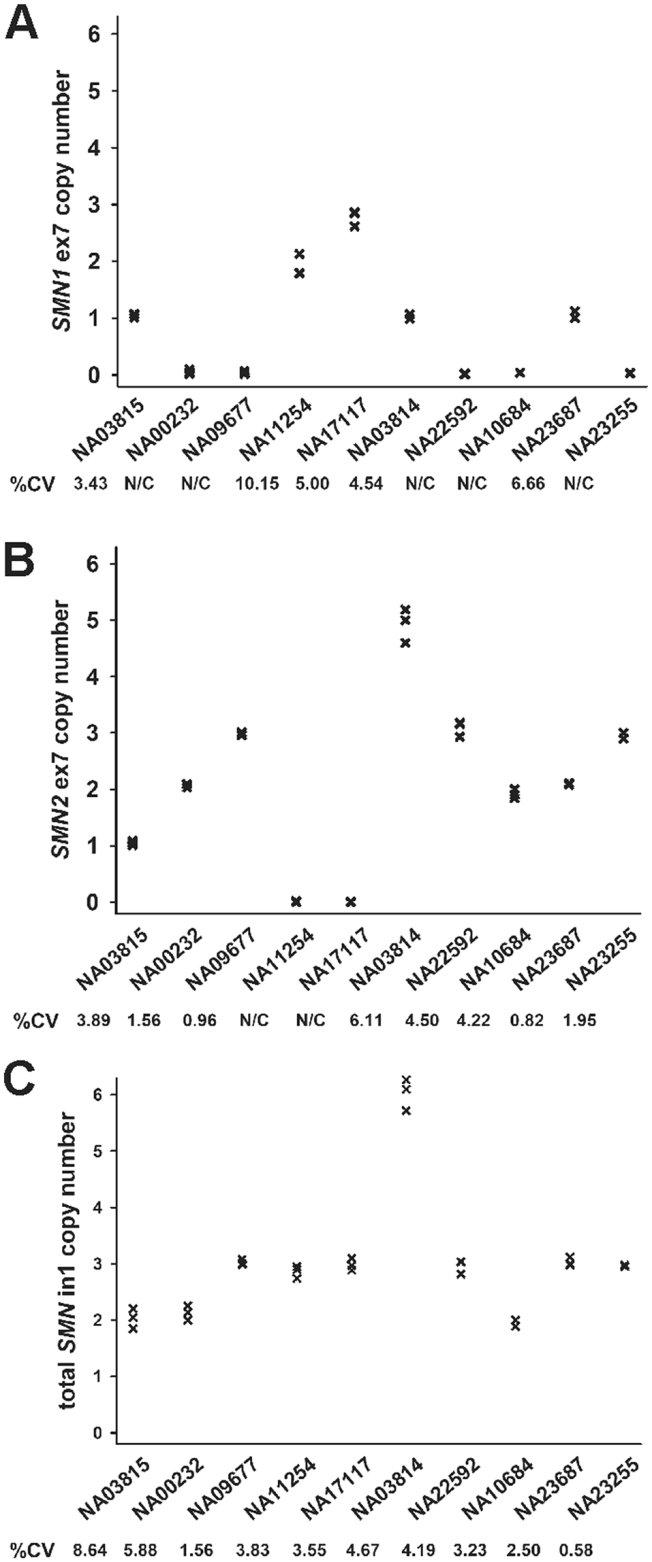
Repeatability of the Absolute Q dPCR assays for SMA genotyping. The control DNA samples (n = 10) from Coriell Cell Repositories were assayed for *SMN1* (**A**), *SMN2* (**B**) and total *SMN* (**C**) copy numbers. Each sample was assayed in triplicate. The intra-assay precision, measured by %CV, for each assay was listed below each sample on the plots. The intra-assay precision measurements were not calculated (N/C) for those samples with a copy number less than 1.00.

Copy numbers of *SMN1*, *SMN2* and total *SMN* in 10 genomic DNA test samples from Coriell Cell Repositories were quantified using the SMA multiplex 4-color assay. All 10 Coriell DNA samples were run in triplicate on the Absolute Q dPCR System for assay verification and repeatability. The intra-assay variability—measured by %CV—between the *SMN1* (Fig. 5A), *SMN2* (Fig. 5B) and total *SMN* (Fig. 5C) is low, demonstrating strong repeatability. The intraclass correlation coefficients (ICCs) were 0.993 (95% confidence interval (CI) 0.979–0.998) for the *SMN1* assay, 0.995 (95% CI 0.985–0.999) for the *SMN2* assay and 0.987 (95% CI 0.963–0.996) for the total *SMN* assay. The *SMN1* and *SMN2* copy numbers measured with this multiplex assay are concordant with those provided by Coriell Cell Repositories, including one sample with 5 copies of *SMN2*. Additionally, the 6 copies of total *SMN* for NA03814 correctly matched the corresponding sums of *SMN1* and *SMN2* copy numbers. The total *SMN* copy numbers in all of the samples except for NA11254 were equal to their corresponding sums of *SMN1* and *SMN2* copy numbers.

### *SMN1* and *SMN2* copy number validation of blinded clinical samples

**Figure 6:**
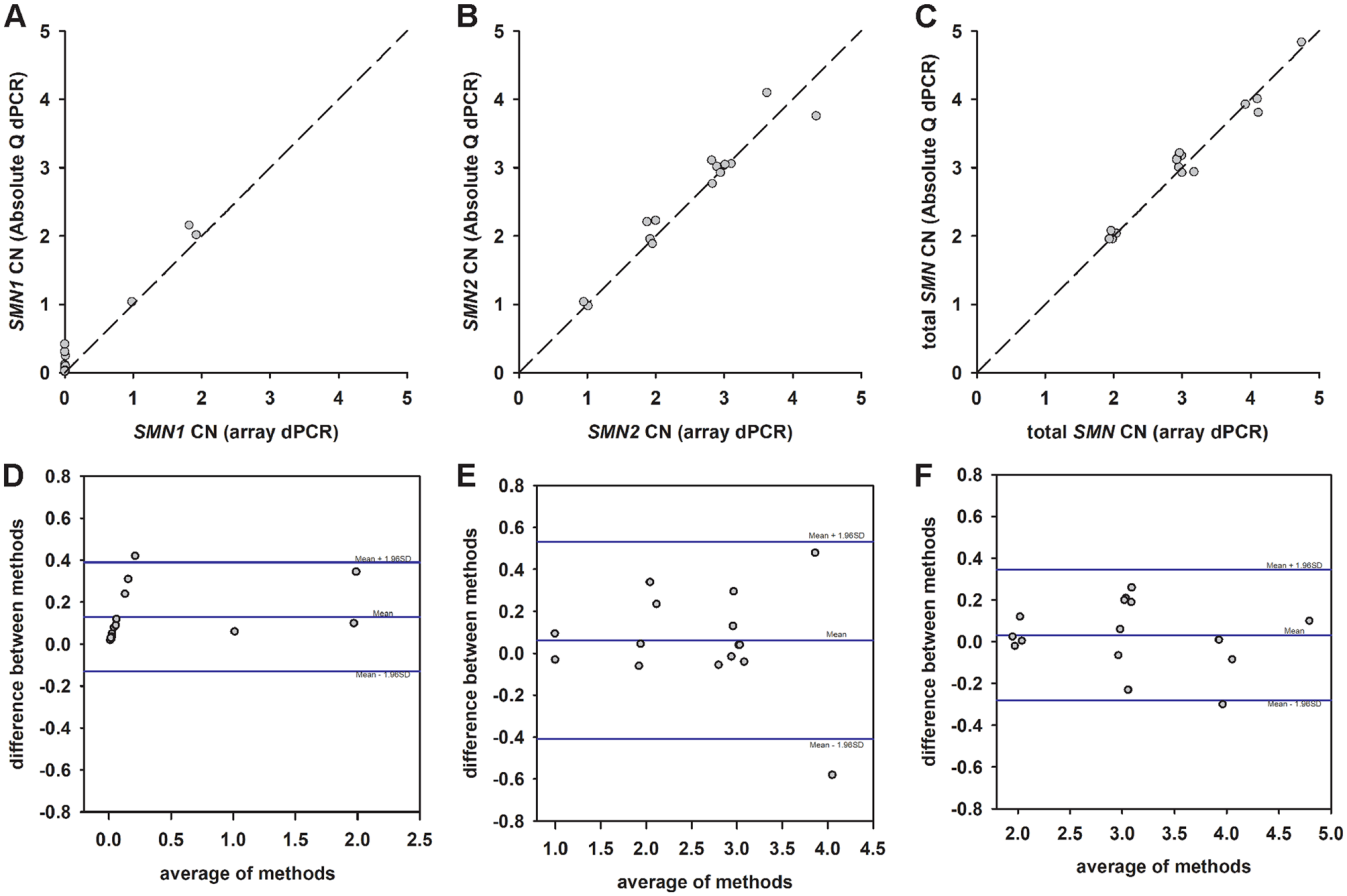
Agreement between the QuantStudio 3D and the Absolute Q dPCR assays. (**A**–**C**) Scatterplots for *SMN1* (**A**), *SMN2* (**B**) and total *SMN* (**C**) copy numbers measured with the QuantStudio 3D and the Absolute Q dPCR assays. The line of equality for each set of assays is shown as a dashed line in each plot. (**D**–**F**) Bland Altman difference plots for *SMN1* (**D**), *SMN2* (**E**) and total *SMN* (**F**) assays. For each assay, the bias is shown as a solid blue line that is labeled as the mean and the limits of agreement defined and labeled as the mean ± 1.96SD (standard deviation).

Absolute Q and QuantStudio 3D array dPCR SMA assays were compared on a set of blinded samples derived from patient lines (n = 15). This set of samples contained gDNAs from 13 SMA patients and 2 non-SMA controls. Using the *SMN1* copy number assay, the Absolute Q dPCR correctly identified all SMA samples with homozygous deletions of *SMN1* (12 out of 15) within the cohort. The Absolute Q dPCR identified one of the patient-derived sample (MND10) as containing 1 copy of *SMN1* and one copy of *SMN2*. This sample was from an individual harboring a *SMN1*(p.A2G) missense mutation^27^. The Absolute Q dPCR *SMN1* (Fig. 6A), *SMN2* (Fig. 6B) and total *SMN* (Fig. 6C) copy number measurements of the blinded clinical samples were concordant with those results obtained using QuantStudio 3D array dPCR^27,31^, including a sample (MND12) with 5 copies of total *SMN*. Bland Altman analysis of the results from *SMN1* (Fig. 6D), *SMN2* (Fig. 6E) and total *SMN* (Fig. 6F and Table 1) probes demonstrated strong agreement between Absolute Q and QuantStudio 3D dPCR assays. For each assay, 93% of the test samples fell within the limits of agreement. Additionally, the total *SMN* copy numbers from 14 blinded clinical samples agree with the sum of the copy numbers from *SMN1* and *SMN2* assays. In a single sample (MND03), the total *SMN* copy number at exon 7 (4 copies) was greater than the sum of the *SMN1* exon 7 (0 copies) and *SMN2* exon 7 (2 copies) copy numbers. Based on this result, this sample was hypothesized to have 2 copies of either *SMN1* or *SMN2* containing a partial deletion of exon 7. The partial deletion of exon 7 in this sample was confirmed orthogonally by junctional PCR (data not shown) and by GS^23^.

**Table 1:** *SMN1* exon 7, *SMN2* exon 7 and total *SMN* intron 1 copy numbers for patient-derived cell lines measured by QuantStudio 3D array dPCR and combination absolute Q dPCR.

Sample	*SMN1* ex7		*SMN2* ex7		*SMN* in1	
	3D dPCR	|Q| dPCR	3D dPCR	|Q| dPCR	3D dPCR	|Q| dPCR
MND01	0	0.08	3	3.02	3	3.18
MND02	0	0.25	3	3.06	3	3.01
MND03	0	0.03	2	1.96	4	3.93
MND04	0	0.12	3	2.93	3	3.14
MND05	0	0.05	3	3.11	3	3.12
MND06	0	0.10	2	2.23	2	2.04
MND07	0	0.02	3	3.04	3	2.94
MND08	0	0.31	4	3.76	4	4.01
MND09	0	0.04	4	4.10	4	3.81
MND10	1	1.04	1	0.98	2	1.96
MND11	2	2.02	1	1.04	3	2.93
MND12	2	2.16	3	2.77	5	4.84
MND13	0	0.02	3	3.05	3	3.22
MND14	0	0.42	2	2.21	2	2.08
MND15	0	0.03	2	1.89	2	1.96

## Discussion

Accurate quantification of *SMN1* and *SMN2* copy numbers is essential for the diagnosis of SMA as well as for the development of therapeutic strategies to treat the disease. This study describes the development of a multiplex dPCR assay that provides accurate and rapid *SMN1* and *SMN2* copy number quantification. The Absolute Q dPCR System provides reliable measurements of *SMN1* and *SMN2* copy numbers with a workflow that is similar to qPCR, reducing hands-on time and time-to-results when compared to other currently available dPCR platforms.

Some dPCR copy number variation assays rely on digestion of the template genomic DNA with a restriction endonuclease^23^. This is because sequence repeats of two or more copies can be in close proximity to each other, increasing the probability that they will be partitioned in the same microchamber and result in a miscalculation of the copy number. Restriction endonuclease digestion of the template DNA helps to reduce the probability of proximal gene copies partitioning in the same microchamber. In this study, *SMN1* and *SMN2* copy numbers were accurately measured without restriction endonuclease digestion of the sample genomic DNA. Each target-specific assay was designed with distinct fluorophores that can reliably differentiate between *SMN1* and *SMN2;* even though these genes share highly homologous DNA sequences. Additionally, the high partition numbers of the microfluidic device (20,000 partitions) and the low DNA input amount (~ 2 to 3 ng per reaction) facilitate single molecule partitioning. Finally, *SMN1* and *SMN2* genes, each approximately 28 kb in length, are both located in the same region of chromosome 5 but they are separated from each other by about 875 kb of genomic sequence. Mechanical sheering of the genomic DNA during DNA sample isolation may further fragment this region thereby reducing the likelihood of both genes being in the same partition. Eliminating the restriction digestion step in the copy number assay, therefore, simplifies the workflow of the assay. Future studies would assess the effects of specimen source and DNA isolation procedure on the need for either a shearing or digestion step prior to dPCR analysis.

In addition to eliminating the need for a pre-PCR digestion step, the dPCR workflow is simplified by integration of multiple systems into a single instrument. The first-generation of dPCR systems have separate instruments for microfluidic partitioning of the PCR reactions and template DNA, PCR amplification and raw data acquisition. This required significant hands-on-time and training for each unique workflow, which, in turn, increased the probability of operator error or process variability. The Absolute Q dPCR System combines sample partitioning, thermocycling, and raw data acquisition into a single benchtop instrument. As a result, the Absolute Q dPCR System has an average run-time that is 73% faster than first-generation dPCR systems^27,31^. The shortened run-time along with minimal hands-on operation could facilitate platform adoption into diagnostic and prognostic settings.

In addition to determining *SMN1* and *SMN2* copy numbers, dPCR can also identify partial deletions in *SMN1*/*SMN2* and gene conversion events that generate hybrid *SMN* genes. By comparing total *SMN* copy number to the sum of *SMN1* exon 7 and *SMN2* exon 7 copy numbers, partial deletions of *SMN1* or *SMN2* can be identified. One of the patient-derived cell lines tested in this study (MND003) contained a partial deletion of exon 7, which was confirmed orthogonally (data not shown). Additionally, one of the control samples (NA11254) possibly contains a partial deletion as the total *SMN* copy number was found to be 3 at intron 1 while the sum of *SMN1* and *SMN2* copy numbers at exon 7 was 2. The discrepant results with this sample are not a consequence of a lack of specificity in the intron 1 primers or probe since the in silico specificity scores for these sequences were high, i.e. all had a BLAT score of 22, which exceeds the minimal threshold of 20^32^. These results need to be confirmed orthogonally by junctional PCR or GS in order to verify that the partial deletion is indeed the reason for the discrepancy. Partial *SMN* deletions, particularly resulting from the loss of exons 7 and/or 8 (*SMN1/2Δ78*), have been previously reported in the literature using several approaches including MLPA and GS^23,33^. Gene conversion events can create hybrid *SMN* genes where part of the gene is *SMN1* and another part is *SMN2*^34^. The most commonly identified hybrid gene is *SMN1* with c.840T (i.e. *SMN2*), however there are other *SMN1*/*SMN2* hybrid gene variations. Other *SMN1*/*SMN2* hybrid genes have different exon 7 inclusion efficiencies compared to *SMN1* or *SMN2*^35,36^. It is important to correctly identify hybrid *SMN* genes as they can potentially lead to unexpected responses to current therapeutic options. Future work involving the development of a panel of assays targeting the single nucleotide difference between *SMN1* and *SMN2* at exon 8, as well as the intronic single nucleotide differences, would be useful for the identification of partial deletions in *SMN* genes as well as hybrid *SMN* genes.

A limitation of the copy number assay described in this study is its inability to detect silent SMA carriers (2 + 0) where both copies of *SMN1* are present in cis on the same chromosome. A single nucleotide polymorphism within *SMN1* (g.27134T>G; rs143838139) is associated with the cis configuration of *SMN1*^37^. In addition, this dPCR assay cannot detect intragenic point mutations in *SMN1,* which account for 3–5% of SMA cases^8^. Future studies are needed to develop dPCR assays that are able to detect these single nucleotide changes. These assays could potentially be integrated into an expanded multiplexing assay panel alongside the current *SMN1*/*SMN2* differentiating dPCR assays described here.

Newborn screening of SMA is becoming more available and routine in the United States as well as many other countries^38,39^. Dried blood spots (DBS) are a common source material for SMA newborn screening. A 3-mm diameter DBS typically yields ~ 140 ng gDNA (in a volume of 50 μL) using column-based DNA extraction^40^. This study demonstrates that *SMN1* and *SMN2* copy numbers can be accurately measured with as little as 2–3 ng gDNA, so the Absolute Q and MAP plates could reliably be used for orthogonal validation of SMA carrier status or *SMN2* copy number. As the technology is further developed, quadraplex dPCR could potentially become a primary SMA screening tool.

The acquisition cost for the Absolute Q dPCR System is comparable to high-end qPCR instrumentation, and somewhat lower than other currently available dPCR platforms. In this study, the consumable (SMA reagents and MAP plates) costs were $2.36 per test. This is less than typical per-sample costs for available droplet dPCR systems ($3.80 per sample^41^) and other technologies such as mass spectrometry ($3.00 per sample^42^). These per-sample costs are exclusive of the cost associated with labor, which is reduced with the lower hands-on time of the Absolute Q dPCR System.

The Absolute Q dPCR quadruplex assay approach could be extended to identification of copy number variations associated with other diseases, in addition to SMA. dPCR has been used to identify rare gene variants as well as differences in copy number of multiple genes associated with pediatric-onset disorders (reviewed in^24^). We have demonstrated that this system can identify a rare single nucleotide variant (*EGFR(T790M)*) associated with non-small cell lung carcinoma as well as fusion genes (*BCR-ABL1* and *CCDC88C-FLT3*) that are associated with different types of cancer^29^.

Accurate quantification of *SMN1* and *SMN2* copy numbers is essential for the proper diagnosis of SMA and the identification of carriers. Similarly, accurate measurement of *SMN2* copy number is essential to determine therapeutic regimens for treating children with SMA^15,16^. Digital PCR—including the Absolute Q dPCR System presented here—can accurately, reliably and rapidly quantify *SMN1* and *SMN2* copy numbers in genomic DNA samples. Future work will assess the feasibility of this dPCR platform for diagnostic as well as prognostic purposes using well-established assay validation approaches and different types of DNA samples^43^.

## Materials and methods

### DNA samples

Control samples of human genomic DNA (n = 10) were obtained from Coriell Cell Repositories (Camden, NJ; Table 2). Most of these samples (n = 8) had well characterized *SMN1* and *SMN2* copy numbers provided by Coriell Cell Repositories. Among the 10 samples, 5 were derived from SMA patients with 0 copies of *SMN1*. The copy numbers for NA11254 and NA17117 were measured using high-resolution melting analysis and array digital PCR (personal communications). NA03814 and NA03815 samples were from known SMA carriers, i.e. 1 copy of *SMN1*.

**Table 2:** Control DNA samples from the coriell cell repositories previously measured *SMN1* and *SMN2* copy numbers for the control DNA samples were obtained from Coriell cell repositories.

Coriell ID	*SMN1 copy number*	*SMN2 copy number*	Description
NA03815	1	1	Known carrier for spinal muscular atrophy
NA00232	0	2	Spinal muscular atrophy type I (SMA1)
NA09677	0	3	Spinal muscular atrophy type II (SMA2)
NA11254	2	0	Ataxia-telangiectasia
NA17117	3	0	Human variation panel control
NA03814	1	5	Known carrier for spinal muscular atrophy
NA22592	0	3	Spinal muscular atrophy type II (SMA2)
NA10684	0	2	Spinal muscular atrophy type I (SMA1)
NA23687	1	2	Known carrier for spinal muscular atrophy
NA23255	0	3	Spinal muscular atrophy type III (SMA3)

Patient-derived samples (n = 15) were procured from the Motor Neuron Diseases Research Laboratory (Nemours Alfred I. duPont Hospital for Children). Genomic DNA was isolated from patient-derived fibroblast and lymphoblastoid cell lines and the *SMN1* and *SMN2* copy numbers were measured using the QuantStudio 3D Digital PCR System (Life Technologies, Waltham, MA) as described previously^27,29^. Approval for cell line generation was provided by the Nemours Institutional Review Board and this study is registered on ClinicalTrials.gov (NCT01754441 and NCT02532244). This study was approved by the Nemours Institutional Review Board (#764456). Written informed consent or assent was obtained for each patient-derived cell line. Each cell line was de-identified. For all samples tested, the tester was blinded to the previously measured *SMN1* and *SMN2* copy numbers. All procedures performed in this study involving human participants were in accordance with the ethical standards of the institutional and/or research committees and with the 1964 Helsinki declaration and its later amendments of comparable ethical standards.

### Absolute Q digital PCR system and microfluidic array partitioning (MAP) plates

The Combinati Absolute Q Digital PCR System consists of Microfluidic Array Partitioning (MAP) consumable plates and a fully integrated instrument that automates partitioning of reagents in the plate, PCR thermocycling and 5-color fluorescence image acquisition. The MAP plate has a standard microplate footprint capable of running up to 16 samples per dPCR run^29^. Each unit is loaded with 10 µL of sample mixture and more than 90% of the loaded sample is partitioned and analyzed in ~ 20,000 pico-scale partitions. The partition volume in MAP plates is defined by the physical dimensions of the microarray chambers and not by a stochastic process such as fluid emulsions, which is important because consistent partition volume is a critical component of the dPCR statistical correction model. The physical array ensures that all samples across all plates yield ~ 20,000 analyzed partitions and minimal sample is lost to dead volume or compromised partitions.

The Absolute Q digital PCR System has a walk-away workflow identical to traditional qPCR. The MAP plate is loaded via pipette with 10 μL of PCR mix and an overlay of 10 μL Isolation Buffer in each well. The wells are then capped with specialized gaskets. The plate is then placed into the Absolute Q tray and retracted into the system. The Absolute Q uses positive pressure from an on-board compressor to partition the sample within the MAP plate without the need for microfluidic valves, sealing films or other moving parts. The MAP slides are constructed of a cyclo olefin polymer (COP; 80 μm) film that seals the microfluidic features that are molded into a separate piece of thicker material. Four identical slides are bonded to a rigid, microtiter format plate frame that includes the loading wells to complete the plate assembly. The thin film becomes gas permeable when positive pressure is applied to a well containing reagents. As the reagent enters the microfluidic features, air is passed out of the partition through the film. This allows reagent to completely fill dead-ended partitions and prevents any bubbles from forming inside of the microfluidic features. The Isolation Buffer overlay follows the reagent and physically separates the reagent reaction volumes to complete the partitioning. Positive pressure applied to the consumable during the PCR thermocycling prevents any evaporation and ensures that bubbles will not form and disrupt the isolated micro-reactions.

Before and after PCR thermocycling, entire arrays are imaged with up to five optical channels configured for the most commonly used dyes, including a ubiquitous quality control dye (ROX) used to verify proper partition filling and finding. The images taken before PCR are subtracted from the after-PCR images to remove background noise. Combinati Analysis software automatically applies optical crosstalk compensation and classifies the partitions using a convoluted neural network algorithm to eliminate false positives/ negatives and ensure robust quantification results. The full dPCR process occurs within the single benchtop instrument without operator’s interaction after setting up the protocol parameters (Fig. 1). The streamlined workflow reduces the potential for contamination, minimizes human handling errors, and reduces the time to result. No fluids ever contact instrument components, so minimal system maintenance is required.

### Digital PCR primer and probe design

The primer and probe oligos were designed using PrimerQuest Tool (Integrated DNA Technologies, Coralville, IA). All primers were checked for target sequence specificity using NCBI Primer-BLAST^44^ and UCSC In-Silico PCR (https://genome.ucsc.edu/, last accessed May 27, 2020) using default settings. For Primer-BLAST, each primer was required to have at least 2 total mismatches to unintended targets, including at least 2 mismatches with the last 5 base pairs at the 3′ end. For In-Silico PCR, the minimum match size was 15 and a minimum BLAT^32^ score of 20, using the GRCh38/hg38 human genome assembly. Primers and probes were also evaluated for primer dimers and cross primer interactions using Multiple Primer Analyzer (Thermo Fisher Scientific, Waltham, MA) with the optimal sensitivity value (3) for dimer detection. All of the oligos were synthesized by Integrated DNA Technologies and Thermo Fisher Scientific. The assay includes a total of 3 pairs of primers and 4 probes with the *SMN1* and *SMN2* targets sharing a common pair of primers. The FAM-labeled *SMN1* and VIC-labeled *SMN2* probes were designed utilizing the signature c.840 single nucleotide difference between exon 7 of *SMN1* (c.840C) and *SMN2* (c.840T). The oligos for total *SMN* copy number were designed upstream in intron 1 targeting both *SMN1* and *SMN2* genes, and the probe was labeled with TYE665. The TAMRA-labeled probe for RNase P (*RPPH1*; OMIM #608513) targeted the single exon region of *RPPH1* gene (Fig. 2). All probes have either QSY or IBRQ dark quenchers on their 3′ ends (Table 3).

**Table 3:** List of primers and probes used for the multiplex dPCR assay.

Name	Description	Oligonucleotide Sequence (5′ > 3′)
SMN1f	Forward primer	5′-AATGCTTTTTAACATCCATATAAAGCT-3′
SMN1r	Reverse primer	5′-CCTTAATTTAAGGAATGTGAGCACC-3′
SMN1p	Probe	5′-FAM-TCCTTACAGGGTTTCAGACAAAATCAA-QSY-3′
SMN2f	Forward Primer	5′-AATGCTTTTTAACATCCATATAAAGCT-3′
SMN2r	Reverse Primer	5′-CCTTAATTTAAGGAATGTGAGCACC-3′
SMN2p	Probe	5′-VIC-TCCTTACAGGGTTTTAGACAAAATCAA-QSY-3′
totalSMNf	Forward primer	5′-GGAAGTTTCAGGAAGTGGTAGG-3′
totalSMNr	Reverse primer	5′-CCACCAGGACTGCCTTTATATC-3′
totalSMNp	Probe	5′- TYE675-AGAAGATGGCAGGGTGTTGGGAAT-IAbRQSp-3′
RPPH1f	Forward primer	5′-CTTTGCCGGAGCTTGGA-3′
RPPH1r	Reverse primer	5′-GAGAGTAGTCTGAATTGGGTTATGA-3′
RPPH1p	Probe	5′-6-TAMRA-ACCTCACCTCAGCCATTGAACTCAC-IAbRQSp-3′

### Digital PCR

The control DNA samples were diluted to 10 ng/µL working stock. The blinded DNA samples were measured using Qubit 4 fluorometer (Invitrogen) and diluted to 10 ng/µL. The PCR reaction mix contained 1X Combinati MasterMix, 2.5–25 ng human gDNA, 1800 nmol/L *SMN1*/*SMN2* primers, 900 nmol/L *RPPH1* primers, 900 nmol/L total *SMN* primers, and 250 nmol/L of each probe. Each MAP plate well was loaded by hand via pipette with 10 µL of PCR reaction mix then overlaid with 10 µL Isolation Buffer. Gasket caps were placed and the plate was put into the Absolute Q and run with the following conditions: 3 min activation at 95 °C, 40 cycles of 5 s at 95 °C and 30 s at 62 °C. The fully automated Absolute Q Control Software (v1.0.20, https://www.combinati.com/) controls the sample digitization into the MAP partitions, thermal cycling, and imaging.

### Data analysis

The copy numbers were determined using the Absolute Q Analysis Software (v10.5.4). The software automatically calculates the optimal positive/ negative threshold and in this case the positive signals were five times higher than the negative signals. For each unit, the software displays the total viable partition count and the positive partition count. The software has a setting to identify the sample types as a copy number variation assay and applies the appropriate calculations to automatically display the copy numbers for the samples. The copy numbers of *SMN1*, *SMN2* and total *SMN* are normalized by the reference control *RPPH1* using the following equations:

$$SMN1 \mathrm{CN}=2 \frac{ln\left(\frac{N}{N-{N}_{1}}\right)}{ln\left(\frac{N}{N-{N}_{4}}\right)}$$

$$SMN2 \mathrm{CN}=2 \frac{ln\left(\frac{N}{N-{N}_{2}}\right)}{ln\left(\frac{N}{N-{N}_{4}}\right)}$$

$$\mathrm{total} SMN \mathrm{CN}=2 \frac{ln\left(\frac{N}{N-{N}_{3}}\right)}{ln\left(\frac{N}{N-{N}_{4}}\right)}$$

where N = total number of viable partitions, N_1_ = number of *SMN1* positive partitions, N_2_ = number of *SMN2* positive partitions, N_3_ = number of total *SMN* positive partitions and N_4_ = number of *RPPH1* positive partitions.

### Statistical analysis

The coefficient of variability (%CV), defined as the standard deviation divided by the mean value for each set of replicates, was used to assess repeatability, or intra-assay precision. Reliability was also measured by the intraclass correlation coefficient (ICC) using SPSS v.25 (IBM, Armonk, NY). A Bland Altman agreement analysis^45^ using SigmaPlot v.12.0 (Systat Software, Inc., San Jose, CA) was used to measure the agreement between dPCR methods.

## Supplementary information

Supplementary Table S1.

## Data Availability

The datasets generated or analyzed during the current study are available from the corresponding author on reasonable request.
