# Supplementary material for: Development and validation of a 4-color multiplexing spinal muscular atrophy (SMA) genotyping assay on a novel integrated digital PCR instrument
Source: Sci Rep. 2020 Nov 16;10:19892. doi: 10.1038/s41598-020-76893-7 (PMC7670453; doi:10.1038/s41598-020-76893-7)
Supplement: Supplementary file 1 — Supplementary Table S1. [file 41598_2020_76893_MOESM1_ESM.docx]

**SUPPLEMENTARY INFORMATION**

**Development and Validation of a 4-Color Multiplexing Spinal Muscular Atrophy (SMA) Genotyping Assay on a Novel Integrated Digital PCR Instrument**

Lingxia Jiang^1,*^, Robert Lin^1^, Steve Gallagher^1^, Andrew Zayac^1^, Matthew E. R. Butchbach^2,3,4,5^ and Paul Hung^1
1^Combinati Inc., Palo Alto, CA; ^2^Center for Applied Clinical Genomics, Nemours Biomedical Research, Nemours Alfred I. duPont Hospital for Children, Wilmington, Delaware; ^3^Center for Pediatric Research, Nemours Biomedical Research, Nemours Alfred I. duPont Hospital for Children, Wilmington, Delaware; ^4^Department of Pediatrics, Sidney Kimmel College of Medicine, Thomas Jefferson University, Philadelphia, Pennsylvania; ^5^Department of Biological Sciences, University of Delaware, Newark, Delaware

*Corresponding author: Lingxia Jiang, Combinati Inc., 2450 Embarcadero Way, Palo Alto, CA, 94303, USA. Email: lingxia@combinati.com

| **Supplementary Table S1 Comparison of Low and High DNA Amount in the dPCR** | | | | | | |
| --- | --- | --- | --- | --- | --- | --- |
| **COMBiNATi Absolute Q dPCR data from gDNA NA23687 at 2.5 ng per PCR** | | | | | | |
| Channel | Name | conc. (cp/ul) | Total partitions | Positive partitions | Measured copy numbers | Expected copy numbers |
| FAM | *SMN1* | 36.75 | 20477 | 364 | 0.98 | 1 |
| VIC | *SMN2* | 76.98 | 20477 | 755 | 2.06 | 2 |
| TAMRA | *RPPH1* | 74.80 | 20477 | 734 | 2-Cp reference | |
| TYE | *SMN* | 113.36 | 20477 | 1102 | 3.03 | 3 |
| **COMBiNATi Absolute Q dPCR data from gDNA NA23687 at 25 ng per PCR** | | | | | | |
| Channel | Name | conc. (cp/ul) | Total partitions | Positive partitions | Measured copy numbers | Expected copy numbers |
| FAM | *SMN1* | 407.62 | 20473 | 3693 | 1.02 | 1 |
| VIC | *SMN2* | 805.16 | 20473 | 6652 | 2.01 | 2 |
| TAMRA | *RPPH1* | 801.01 | 20473 | 6624 | 2-Cp reference | |
| TYE | *SMN* | 1184.92 | 20473 | 8990 | 2.96 | 3 |
